# International Medical Congress

**Published:** 1887-06

**Authors:** 


### International Medical Congress.

The preliminary work in all the departments of the Congress is progressing very satisfactorily. Of the preparation for the Dental Section we can speak more definitely. Papers to be read before the section are rapidly coming into the hands of the secretary; and are being arranged and prepared for proper presentation before the section. The various committees are giving active attention to their respective duties. A large number of the best members of the profession in this and foreign countries have engaged to give clinics in operative and surgical dentistry, and demonstrations in the prosthetic department; and doubtless the best talent of the world, in these branches, will be present. Opportunity will be afforded to see what the microscope has done for dentistry. A microscopic department is being provided for, in which will be presented the achievements of the microscope, by means of a large exhibit of preparations as well as the various methods of mounting objects for observation.

By the energy and perseverence of the local committee in Washington, large and ample accommodation in the way of rooms, etc., has been secured for all the work of the section. For this the committee will have the lasting gratitude of all concerned, and we hope even more substantial recognition than this. A very commodious room for the general meetings of the section has been secured in a church; all these rooms are in the same locality, or sufficiently so for convenience, and they are all within a reasonable distance of the principal hotels. The Arlington Hotel will be the headquarters for the Dental Section. It will be well for all who desire good quarters during the meeting to write and secure rooms at an early day. Some reduction of rates will be made by all the hotels. The committee on transportation have secured rates from several steamship lines as follows: from Liverpool to New York and return, $80 to $95, for first-class accommodations.

The various railroads of this country will doubtless give reduced rates to all who attend the Congress.

